# Carbon Pathways Through the Food Web of a Microbial Mat From Byers Peninsula, Antarctica

**DOI:** 10.3389/fmicb.2019.00628

**Published:** 2019-03-28

**Authors:** Pablo Almela, David Velázquez, Eugenio Rico, Ana Justel, Antonio Quesada

**Affiliations:** ^1^Biology Department, Autonomous University of Madrid, Madrid, Spain; ^2^Ecology Department, Autonomous University of Madrid, Madrid, Spain; ^3^Mathematics Department, Autonomous University of Madrid, Madrid, Spain

**Keywords:** microbial mats, Antarctica, cyanobacteria, trophic web, carbon pathways, stable isotopes, prokaryotic community, eukaryotic community

## Abstract

Microbial mats are complex communities that represent a large biomass fraction in non-marine Antarctic ecosystems. They confer structure to soils and constitute, by themselves, intricate microecosystems, where a great variety of microorganisms and microfauna contributes to the ecosystem functions. Although in recent years Antarctic microbial mats have been thoroughly investigated, trophic relationships within the communities remain unresolved. We therefore conducted a study of the trophic relationships of a microbial mat from Byers Peninsula, Antarctica, using DNA analysis and stable isotopes as trophic tracers. Our results suggested, based on a Bayesian mixing model, that at least four trophic levels are present within this microecosystem: primary producers (cyanobacteria and diatoms), primary consumers (rotifers and tardigrades), secondary consumers (nematodes) and decomposers (fungi). Nematodes would play a key role as top consumers of the community, connecting the two carbon inputs described into the system, as omnivores at the secondary trophic level. In addition, carbon pathways from primary trophic level to consumers take place quickly during the first 24 h after its incorporation in the primary producers, dispersing across all the trophic levels and reaching secondary consumers in less than 11 days. This suggests that, given the changing physical conditions and presumably short periods of activity, there is a fine temporal coupling among the organisms in the community, minimizing the redundancy in function performance among trophic levels.

## Introduction

Microbial mats, with a ubiquitous distribution throughout Antarctica, are the most widespread microbial consortia in terrestrial landscapes. They constitute the largest non-marine biomass concentrations in these regions (Quesada et al., 2008) and accumulate the greatest biodiversity in inland waters, being recognized as hotspots for biological productivity and diversity. The organisms that inhabit these microecosystems range from viruses to green algae, rotifers, diatoms, nematodes and tardigrades, with cyanobacterial species as the most common organisms (Vincent, 2000; Jungblut et al., 2005). A regular feature in these microecosystems is the presence of differently colored layers due to the different pigmentation of phototrophic microorganisms (Vincent et al., 1993), resulting in a layered vertical structure. These cyanobacteria-based ecosystems have shown a considerable level of community stability throughout time, with some structures almost unchanged over the last 100 years (Jungblut and Hawes, 2017), which suggests a resilience capacity that assures its function as refugia for biological diversity (Chown et al., 2015).

Previous works on microbial mats from polar regions have mostly focused on structural aspects, biodiversity of microbial community and their relationship with the environment (de Los Ríos et al., 2004; Jungblut et al., 2012), but little is known about trophic relationships within these microbial mat ecosystems (Velázquez et al., 2017). Freshwater food webs in Antarctic regions are simpler than those in temperate regions (Hansson and Tranvik, 2003). Soil fauna biodiversity is reduced to 1.1–2.6% of temperate soils (Freckman and Virginia, 1997), and this is also reflected in the complexity of trophic relationships. However, some microbial ecosystems from Antarctica showed larger biodiversity than the same ecosystems from lower latitudes (López-Bueno et al., 2009).

Byers Peninsula is one of the largest ice-free areas in the Antarctic Peninsula region, with a well-developed network of water bodies, especially during the snow-melting season. It has been described as one of the main Antarctic hotspots of biodiversity (Convey et al., 1996; Toro et al., 2007) and proposed as a key observing spot to monitor the effects of climate change on freshwater and terrestrial ecosystems (Quesada et al., 2009). Microbial mats are extremely abundant in Byers Peninsula, particularly in the central plateau and associated to its large freshwater network (Toro et al., 2007). These micro-ecosystems are, therefore, essential to understand the diversity, the community structure and dynamics of these ecosystems to forecast future biological responses to perturbations as, e.g., climate change, human activity or invasive species (Velázquez et al., 2017).

Previous analyses indicate different structural organization of microbial mats depending on their community composition (de Los Ríos et al., 2004; Velázquez et al., 2017). Moreover, community carbon assimilation diverges according to relative abundances of chlorophytes and cyanobacteria (Velázquez et al., 2011), with green algae adapted well to cold temperatures and cyanobacteria performing better in warmer conditions. The present study tests the trophic position and the trophic relationships among living organisms that shape the structure of a cyanobacterial microbial mat from Byers Peninsula during the austral summer, by using the stable isotopes of N and C. Accumulation rates of nitrogen isotope can be used to estimate the trophic position of the organisms because of the differential isotopic enrichment of the organism depending upon its diet (Peterson and Fry, 1987), while ^13^C isotope can be used to describe the origin of the incorporated C. Moreover, there is a natural isotopic discrimination against ^13^C and in favor of ^12^C (Post, 2002), so the trophic relationships (e.g., C transfer) among primary trophic level and consumers in a microbial mat can be determined by incubating the community with δ^13^C enriched substrates. Nematoda, Tardigrada and Rotifera, the main microfaunal groups in Antarctic soils (Sohlenius et al., 2004), were the studied consumers, besides the main primary producers presented during the study.

All the community components were sampled at different time points to track the carbon pathways along the trophic web. To the best of our knowledge, this “*enriching and hunting*” methodology has never been used for microbial mats at these high-latitudes. These results were analyzed and tested by a Bayesian mixing model and completed by a small sub-unit of the RNA (SSU RNA) meta-barcoding approach of the community to characterize the bacterial and eukaryotic populations that might interplay along the trophic web.

## Materials and Methods

### Study Site and Sampling

**FIGURE 1:**
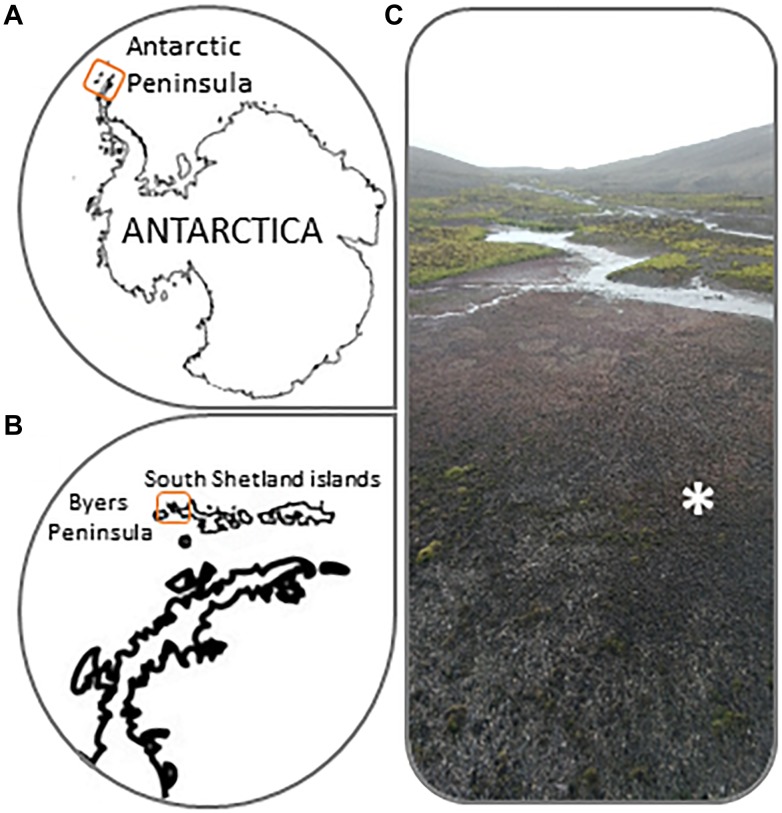
**(A,B)** Location details of Byers Peninsula at Livingston Island in the South Shetland Islands, Antarctica. **(C)** Picture showing common microbial mat types from Byers Peninsula. The symbol “^∗^” indicates where exactly enrichment experiments were conducted.

Sampling was conducted on a microbial mat from Byers Peninsula (Figure 1) during the austral summer in January 2013. The study site is located at the western end of Livingston Island, Antarctica (South Shetland Islands; 62°34′35′′, 61°13′07′′W). Byers Peninsula, with a central plateau length of 18.2 km from northwest-southeast, is the largest ice-free area of South Shetland Islands. It is an Antarctic Specially Protected Area (ASPA N° 126), because of the importance of their biological communities and its geological and archeological values.

The microbial mat was sampled in a flooding area (2–4 cm deep) at the Southern beaches of Byers Peninsula, which was almost 100% covered by microbial mats (Figure 1C). It displayed a vertical structure of bi-layered microbial mats that has been previously described in this area by Rochera et al. (2013), due to the pigmentation of the different layers. Six samples were haphazardly collected with metal spatulas from different areas of the wetland to cover a sampling area of ca. 10 m^2^. These six samples were placed in the experimental trays (about 600 cm^2^) and the sampling within each experimental tray was haphazardly made again. The “enriching and hunting” experiment, explained below, was carried out *on-site* using the collected samples. Replicates of all samples were stored in Whirl-pak bags at -20°C until further analysis at the laboratory.

### Microscopic Identification of the Community Composition

The composition of the community involved in the food web of the microbial mat, grouped in primary producers and consumers (microfauna), was studied using a stereozoom microscope (Leica MZ75). Photosynthetic organisms’ observations were conducted under white illumination and using epifluorescence microscopy with an Olympus^®^ blue filter set (EF 400–490 nm, DM 570, FB 590) where chlorophyll-*a* was excited, while differential cyanobacterial phycobilins were excited with an Olympus^®^ green filter set (EF 530–545 nm, DM 570, FB 590).

### Biomass Determination

Estimations of relative abundances of the microbial mat trophic community were calculated from total biomass from the ash free dry weight (AFDW). After obtaining the carbon content per surface unit of each of the studied groups (consumers, primary producers and fungi), as described below, the biomass value was subtracted from the total carbon calculated for the microbial mat and expressed as percentage.

Phototrophic community biomass (cyanobacteria, green algae, and diatoms) was estimated by chlorophyll-*a* (Chl*a*) concentration and assuming a proportion of Chl*a* per wet weight unit (ww) of 0.9% (Kasprzak et al., 2008). Chlorophyll-*a* was extracted in triplicates from 7 mm in diameter and 2–3 mm in thickness core samples using ethanol, according to European Union standard ISO 10260, which prescribes 90% (v/v) ethanol for chlorophyll extraction and measurement at 665 nm. Extracts were measured using a Hitachi U2000 spectrophotometer. A specific density of 1 g cm^-3^ was assumed for transforming the biovolume of the phototrophic community into dry weight (Wieser, 1960). Carbon content was estimated as 40% of the dry weight, as determined by Feller (1988).

Ergosterol, a biochemical marker of fungal active biomass, was quantified using HPLC equipped with a UV detector (282 nm). For ergosterol extraction cores (7 mm in diameter and 2–3 mm thick according to microbial mat thickness) were placed in triplicates in 12 ml glass culture tubes then 10 ml of KOH 0.14 M were added. Tubes were sealed with rubber caps and placed in a temperature-controlled bath (80°C) for 30 min while shaking. After cooling at room temperature, two additional extractions were carried out with 10 ml methanol and sonication cycles to maximize the ergosterol retrieval. Extracts were mixed and eluted with a 1 ml min^-1^ flow through the solid-phase extraction cartridges (ExtraBond Cartridge C18 1000 mg. Scharlab). The ergosterol in the eluted product was quantified by HPLC, as described by Gessner (2005). The concentration of ergosterol determined by HPLC is transformed to fungal biomass assuming that 5.5 mg of ergosterol are found in 1 g of fungal biomass (Gessner and Chauvet, 1993). Finally, C in the fungal biomass was estimated as 43% of the dry weight (Baldy and Gessner, 1997).

Relative abundance of consumers (rotifers, tardigrades and nematodes) was estimated by direct counting in core samples and interpreted per surface unit. Total biomass of each consumer was estimated measuring length and width from microscope photographs using the image analysis system SigmaScan Pro 5, following the approaches proposed by Ruttner-Kolisko (1977) for rotifers, Benke et al. (1999) for tardigrades, and Burgherr and Meyer (1997) for nematodes. A specific density of 1 g cm^-3^ was assumed for transforming biovolume into dry weight (Bottrell et al., 1976). The dry weight was converted into carbon content, which was assumed to be 40% of the dry weight (Feller, 1988).

### ^13^C and ^15^N Natural Abundance Analysis and Microbial Mat Enrichment Incubations

At the laboratory, microbial mat samples were manually disaggregated, and microorganisms were sorted using a stereozoom microscope (Leica MZ75) for δ^13^C‰ and δ^15^N‰ natural abundance signal analysis. Individuals of different trophic levels were manually separated under the microscope by microdissection and encapsulated in triplicates in 175 pl zinc cases and dried at 65°C for 48 h. A minimum of 0.02 mg of dry weight biomass was needed (Shaw et al., 2018), achieved by collecting approximately 80–100 live individuals per zinc case. Particulate organic matter (POM) between 30 and 0.22 μm was separated by sequential filtering and dissolved organic carbon (DOC), considered as the supernatant <0.22 μm, were analyzed for δ^13^C‰ and δ^15^N‰ as above. The sequential filtration was determined by triplicate as follows: a piece of mat was pressed gently against a Nytal sieve of 30 μm pore size diameter, the filtrate was filtered again through a 5 μm filter and the filtrate was then filtered through 0.5 μm pore membrane. The matter retained in the filters was manually recovered for isotopic analyses. The 0.5 μm filtrate was then filtered through a 0.22 μm hydrophilic membrane and concentrated by evaporation at 40°C under vacuum. Samples were analyzed by a mass spectrometer of isotopic ratios (MEIR; 20-20 PDZ Europa mass spectrometer, Sandbach, United Kingdom).

Stable isotopes of carbon were also used as tracers of the food web, providing information about matter transfers within the microbial mat by an on-site “enriching and hunting” experiment. The community was labeled with ^13^C (98% ^13^C. Isotec) by exposing the community under the sunlight to NaH^13^CO_3_ which was photoassimilated by the primary producers. The incubations were launched by adding the ^13^C tracer to the microbial mat inside the experimental trays at an approximate concentration of 10% of the natural concentration of dissolved inorganic carbon (DIC). DIC was estimated from water alkalinity considering pH and temperature and measured after titration with HCl using phenolphthalein as pH shift double indicator. After an incubation period of 24 h *in situ*, autotrophic organisms, mostly photoautotrophs, should have assimilated the isotope, and excess of unassimilated isotope was removed as described in Velázquez et al. (2017). Then, microbial mat in incubation trays were placed again at the wetland, fixing them to the ground so to avoid water exchange with the surrounding wetland. Samples were obtained at time periods of 0, 8, 24, 48, and 168 (7 days) and 264 h (11 days), with sterile 7 mm diameter brass cylinders from the incubation trays and kept frozen until the organisms were manually separated under stereozoom microscope. The different compartments of the community were encapsulated as described previously for natural abundance analysis, at the different time-lapses previously mentioned. The capsules were analyzed by a MEIR (20–20 PDZ Europa mass spectrometer, Sandbach, United Kingdom). So, carbon isotopic ratios were measured in the different ecological compartments of the community throughout the study period, thus determining the trophic relationships between them by Stable Isotope Analysis in R (SIAR; Parnell et al., 2013) (see below). Stable isotopes enrichment of the fungal community, as an ecological compartment of the food web, could not be finally analyzed due to the small amount of biomass recovered.

### Data Analysis and Modeling

Trophic pathways within the community were studied using a Bayesian isotopic mixing model, available as an open source R package, SIAR (Parnell et al., 2013). The main carbon sources of the studied consumers were determined by comparison between their ^13^C signatures to the groups previously identified as sources. Nematodes were compared to the rest of the community, including rotifers and tardigrades as sources, due to their variety of feeding strategies described by Shaw et al. (2018). The fungal community was considered as decomposers, and was analyzed with those groups considered trophically related and closest after natural abundance analyses.

Also, we assumed similar stoichiometry for C and N fractionation through the food web to implement SIAR modeling. A quadratic function was fitted with data obtained from ^13^C‰ enrichment experiment at different time intervals. Trophic enrichment factors (TEF) were based on mean trophic fractionations with standard deviations that are considered global averages (TEF 5^13^C – 0.4 ± 1.3; TEF 5 ^15^N–3.4 ± 1) (Post, 2002). No parameters were modified between consumers according to their trophic habits, assuming that each group was composed of different species with different characteristics.

### 16S and 18S rRNA Analysis

Bar-coding profiles using high-throughput sequencing were carried out to better assess the microscopic identification of the community composition. Total genomic DNA was extracted separately from three microbial mat cores using the MoBio PowerBiofilm DNA extraction kit (Carlsbad, CA, United States) following the manufacturer’s instructions. The 16S rRNA gene was amplified by PCR using barcoded primers set 27F (5′-AGAGTTTGATCCTGGCTCAG-3′) and 534R (5′-ATTACCGCGGCTGCTGG-3′) targeting the V1–V3 hypervariable regions, according to Crits-Christoph et al. (2013). This universal primer set is for bacterial community and the archaeal community was not included in the study. For 18S rRNA gene, genomic DNA was amplified according to the protocol of Bates et al. (2013), using the eukaryotic-specific primer set 515F (5′-GTGCCAGCMGCCGCGGTAA-3′) and 1119R (5′-GGTGCCCTTCCGTCA-3′), targeting the V4 region. The pool of samples, three for each targeted gene, with the prepared libraries was sequenced by Illumina MiSeq platform.

High-quality sequences were trimmed by “cutadapt” (Martin, 2011) to 300 bp on average and then checked for chimeras using UCHIME (Edgar et al., 2011). Chimeras were discarded for downstream analyses. Using the same rationale, in the case of 18S rRNA results, sequences lower than 150 nts were removed from the analysis. Operational Taxonomic Units (OTUs) were delineated based on 97% sequence by using MOTHUR (Schloss et al., 2009). BLASTN search (Altschul et al., 1990) to SILVA reference database for 16S and 18S rRNA gene sequences was performed to determine the closest cultured and uncultured match. Sampling effort was assessed by calculation on rarefaction curves. Sequences generated by this study were deposited to GenBank under the BioSample accession number SAMN10505289.

## Results

### Community Relative Abundances

Relative abundances (Table 1) were calculated from total biomass of the microbial mat, estimated in 6667 μgC cm^-2^. From there, and for each studied group of the community, relative abundances calculated were: primary producers (8.8%, equivalent to average 587 μgC cm^-2^), fungi community (0.9%, equivalent to average 55 μgC cm^-2^), and consumers (2.4%, equivalent to average 159 μgC cm^-2^). For tardigrades, rotifers and nematodes, relative abundance was estimated in 1.8% (equivalent to average 122 μgC cm^-2^), 0.001% (equivalent to average 0.04 μgC cm^-2^) and 0.6% (equivalent to average 37.2 μgC cm^-2^), respectively. The remaining portion not assigned to any of the studied trophic groups, was named as “remaining biomass,” reaching 87.9% of total estimated carbon.

**Table 1:** Carbon relative abundances of biological compartments from the studied microbial mat (Byers Peninsula, Antarctica).

	μg C/total C	% C/total C
Primary producers	587 ± 91.2	8.8
Fungi	55 ± 4.3	0.9
Nematodes	37 ± 0.1	0.6
Rotifers	0.04 ± 0.1	0.001
Tardigrades	122 ± 0.1	1.8
Remaining biomass		87.9
Total biomass	6667 ± 1886	100


*From total biomass, calculated by AFDW, the carbon amounts represented by each of the organisms were subtracted, finally leaving the percentage of remaining biomass. Primary producers are represented by the photosynthetic organisms (algae and cyanobacteria). Consumers community are represented by tardigrades, nematodes and rotifers. Decomposers community is only represented by the fungi.*

### 16S and 18S rRNA Amplicon Sequencing

Approximately 400,000 valid sequences were obtained, clustered in 1.121 OTUs. OTU richness for Bacteria was 979 OTUs obtained from the V1–V3 hypervariable region of the 16S rRNA gene, and for Eukarya 142 OTUs from V4–V5 region of the 18S rRNA gene. Rarefaction curves indicate that almost the plateau of detection of the bacterial and eukaryotic OTU diversity has been reached (Supplementary Figure 1), so an increase in the number of sequences will not impact the number of OTU detected.

The bacterial community was dominated by Proteobacteria, with 39.4% relative abundance, followed by Bacteroidetes (27.8%), and Cyanobacteria (11.1%). The sequences assigned to Betaproteobacteria were dominated by members of the Burkholderiales (55.2%) order, while Micrococcales (60.3%) and Flavobacteriales (53.2%) were the most abundant for Actinobacteria and Bacteroidetes OTUs, respectively. The cyanobacterial fraction was dominated by Nostocales, with a relative abundance of 8.3%, followed by Oscillatoriales (1.9%) and Chroococcales (0.4%). For eukaryotic community, the group with a relatively greater abundance of OTUs was Chlorophyta (19.4%), followed by Ciliophora (13.8%), Bacillariophyta (11.9%), Cercozoa (10.5%), and Heterokontophyta (6.4%). Fungi-related OTUs (mainly Ascomycota and Basidiomycota-affiliated ribotypes) represented on average 13% of the eukaryotic datasets. Nematoda, Tardigrada and Rotifera showed relative abundances of 2.7, 2.3, and 1.4%, respectively.

**FIGURE 2:**
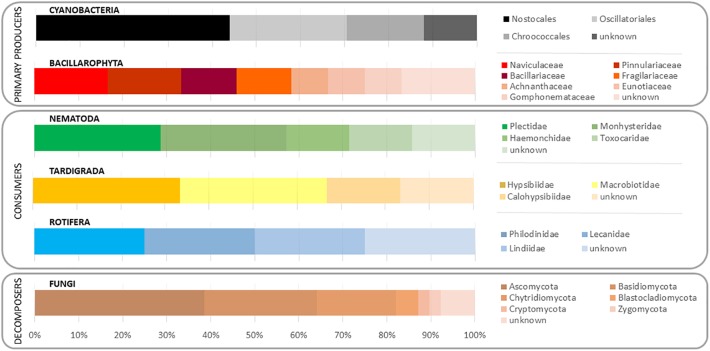
Relative abundance of the studied taxa, including microbial and eukaryotic community, in a microbial mat from Byers Peninsula (South Shetland Islands, Antarctica).

As the diversity of the studied trophic groups was the main interest of the study, their relative abundances per families were scaled to total number reads per phylum (Figure 2). Cyanobacteria was dominated by Nostocaceae (44.0%), followed by Oscillatoriaceae (26.5%) and Chroococcaceae (17.6%). Bacillariophyta’s OTUs matched Naviculaceae (16.2%) and Pinnulariaceae (15.9%), followed by Bacillariaceae (12.7%) and Fragilariaceae (12.0%). The nematode community was dominated by Plectidae (27.0%) and Monhysteridae (25.2%). The predominant families for tardigrades were Hypsibiidae (34.1%) and Macrobiotidae (33.4%), especially the genera *Macrobiotus*, *Isohypsibius*, *Acutuncus*, and *Calohypsibius*. Rotifer community was dominated by OTUs matching Philodinidae, Lecanidae, and Lindiidae families, with very similar relative abundances. Fungal community, which represents an important portion of the decomposers fraction, was dominated by Ascomycota (38.5%) and Basidiomycota (25.6%) OTUs.

### δ^13^C‰ and δ^15^N‰ Natural Abundances of the Community

According to δ^13^C‰ and δ^15^N‰ natural abundance analysis of the studied groups and their ecology, a classification into three different trophic categories was established. Therefore, primary producers, the consumers category, and fungi community as decomposers, were defined. Primary producers included cyanobacteria and diatoms, while consumers category consisted of tardigrades, rotifers and nematodes.

**FIGURE 3:**
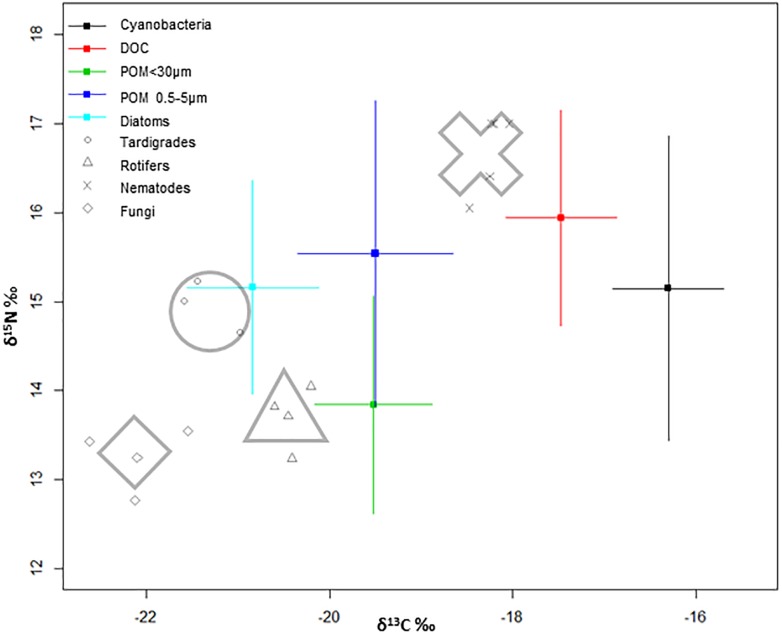
Bivariate model plot of isotopic signatures (δ^13^C and δ^15^N), of organisms in the community and potential food sources of a microbial mat from Byers Peninsula, Antarctica. For sources, points are mean trophic enrichment factor, and error bars are ±1 standard deviation of the trophic enrichment factor. For consumers, shapes around the plots represent the average of each group.

The isotopic δ^13^C‰ signatures of primary producers (Figure 3) were -17.30 ± 0.3 for cyanobacteria and -21.84 ± 0.9 for diatoms, whereas δ^15^N‰ signals were 11.75 ± 0.6 and 11.76 ± 0.1, respectively. DOC isotopic values (-18.5 ± 0.1δ^13^Cvpdb and 12.5 ± 0.1 δ^15^Nair) were assumed to reflect cyanobacterial exudates and/or dissolved exopolymeric substances (EPS). POM smaller than 30 μm includes chlorophytes, partially decomposed matter and some ciliates (-20.5 ± 0.1δ^13^Cvpdb and 10.4 ± 0.1δ^15^Nair). The POM fraction between 0.5 and 5 μm (-20.5 ± 0.3δ^13^Cvpdb and 12.1 ± 0.6δ^15^Nair), which includes a fraction of microbes of the microbial mat, due to the size range filtered, has been plotted above the larger fraction of POM, with a ^13^C/^12^C ratio 2% higher.

Fungi δ^13^C‰ displayed the most negative values within the food web. Tardigrades appear midway between the fungi and rotifers regarding to carbon ratios, and with similar values to those shown by diatoms. Rotifers appear around POM fraction smaller than 30 μm and below tardigrades regarding to nitrogen ratios. In nematodes, there seems to be a tendency toward less positive values of δ^13^C‰ that suggest differences in carbon sources, and toward more positive nitrogen ratios in comparison with the other studied consumers (Figure 3).

### Carbon Tracking Among Primary Producers, Consumers and Decomposers

**FIGURE 4:**
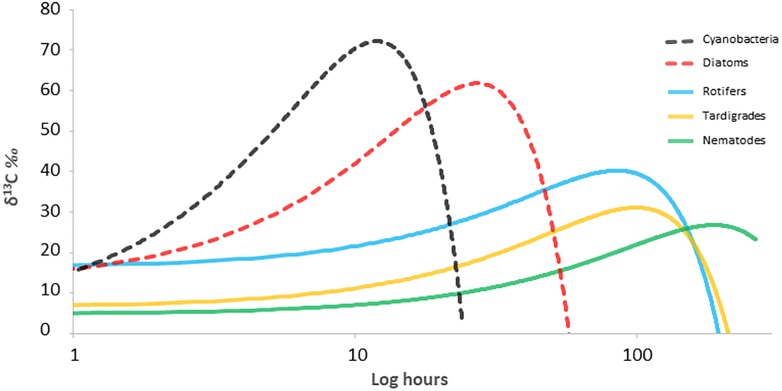
^13^C signal through the different organisms along time in a microbial mat from Byers Peninsula, Antarctica. Lines represent ^13^C data adjusted to a quadratic function. Dashed lines represent primary producers and solid lines consumers.

The circulation of C through different trophic levels was determined from changes in ^13^C/^12^C signatures through the food web at different time points (Figure 4). Cyanobacteria and diatoms showed similar behavior, incorporating the labeled inorganic carbon during the first 24 h after the incubation. The enrichment peak appeared earlier in cyanobacteria, with of 64.8 ± 56.8 δ^13^C‰ for cyanobacteria and 59.1 ± 33.1 δ^13^C‰ for diatoms. Then, δ^13^C‰ values decreased gradually for both, due to the natural predominance of ^12^C in the ecosystem and its incorporation, diluting and re-establishing the initial δ^13^C‰ in the next 2 days. Rotifers and tardigrades were the first consumers that incorporate the labeled inorganic carbon. Rotifers showed the enrichment peak earlier than tardigrades, with values of 33.8 ± 10.6 δ^13^C‰ and 22.9 ± 14.9 δ^13^C‰, respectively. As for primary producers, δ^13^C‰ decreased gradually for both along the experiment. Nematodes showed a gradual enrichment of ^13^C since the beginning of incubation, reaching the highest δ^13^C‰ at 168 h, becoming the last organism of the food web to assimilate the added inorganic carbon. At the end of the study, nematodes δ^13^C‰ signal values continued almost at the same level than from the enrichment peak.

### Food Web Modeling

**FIGURE 5:**
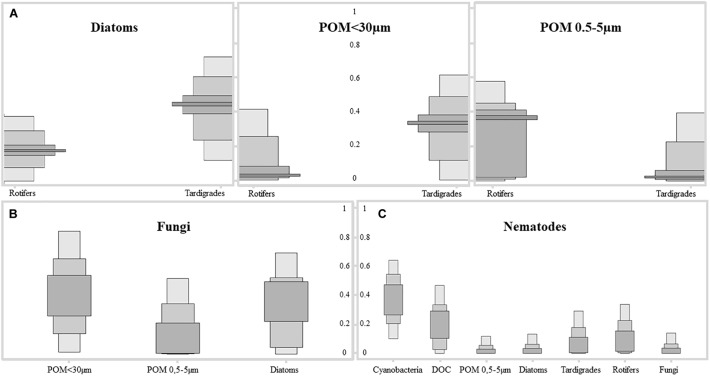
Results of SIAR Bayesian mixing model showing the contribution of each C source to the different fractions of the microbial mat community from Byers Peninsula, Antarctica. Different gray colored boxes indicate confidence intervals of 25, 75, and 95%. **(A)** Results for tardigrades and rotifers by their main sources diatoms, Particulate Organic Matter fraction <30 μm and POM fraction between 0.5 and 5 μm, of the microbial mat community. **(B)** Results for fungi by their main C sources diatoms, POM fraction <30 μm and POM fraction between 0.5 and 5 μm. **(C)** Results for nematodes by their main C sources, where the entire community was included as potential C source.

Stable Isotope Analysis in R model interpretation displayed important trophic interactions between tardigrades and rotifers to diatoms (52 and 36%, respectively), POM <30 μm (35 and 5%) and POM 0.5–5 μm (4 and 35%), respectively, and practically not related to cyanobacteria and DOC (Figure 5). So, a deviation of the tardigrades toward largest POM fraction related to green algae of the microbial mat can be observed while the rotifers would be related to POM fraction of smaller size. Carbon sources of fungal fraction were influenced mainly by POM <30 μm (37%) (Figure 5). Nematodes were included in the model as omnivores, at the top of the food web, including consumers as available C sources. In this case food sources were more dispersed (Figure 5), appearing trophically closer to cyanobacteria (42%) and DOC (21%), but also to tardigrades (7%) and rotifers (10%).

## Discussion

### Community Structure

The high diversity of microbial communities inhabiting Antarctic soils (Barrett et al., 2006) is well known. Benthic microbial mats appear as aggregated microecosystems favoring the establishment of high diversity and complex communities because of the highest nutrient availability and protection against the harsh environmental conditions (De Los Ríos et al., 2014; Chown et al., 2015). Understanding trophic structure of microbial mats from Antarctica is critical to studies of biotic interactions and therefore ecosystems functioning, due to the important role they play in biomass accumulation and productivity. For the first time, we have carried out an *on site* enrichment experiment of an Antarctic microbial mat with ^13^C, that has provided insights into their trophic interactions, together with natural abundance ratios of C (^13^C/^12^C) and N (^15^N/^14^N). The stable isotope composition of microbial mat’s compartments revealed a food web with at least four trophic levels: a basal level of primary producers divided into diatoms and cyanobacteria as different organic carbon inputs, a secondary level of consumers composed of rotifers and tardigrades, then nematodes occupying a higher trophic position, and a 4th level where fungi would act as part of the decomposer’s community. POM less than 30 μm measured in this study contained a mixture of primary producers and detritus.

### Trophic Interactions

Diatoms and cyanobacteria displayed different isotopic signatures (Figure 3) with different C assimilation ratios along the incubation period (Figure 4). It has been considered that cyanobacteria success at high latitudes is due to their wide range of tolerance to conditions and to maintaining slow but constant growth rates, despite the frigid ambient temperatures (Taton et al., 2003; Quesada and Vincent, 2012). However, we have seen how they are metabolically comparable to photosynthetic eukaryotes as diatoms, considered as crucial in colonization and in primary and secondary succession processes (Rahmonov et al., 2015). Therefore, cyanobacteria could be competing at the same level as the other primary producers within the microbial mat community, with comparable photosynthetic efficiency, which is relevant in a cyanobacteria-based microecosystem where they also provide structural integrity to the microbial mat.

The secondary trophic level is composed by rotifers and tardigrades, with similar natural abundance of isotopic composition between each other, but differences in trophic preferences. Results extracted from trophic modeling of the community showed that tardigrades are more trophically related to diatoms and to POM <30 μm than to cyanobacteria (Figure 5). Microbial mat community progressively adjusts its photosynthetic metabolism to environmental conditions (Velázquez et al., 2011), and it is during the initial stages of the spring period when green algae have an important role as a carbon source to the system. During this period, melting occurs from soil to the upper layer of the microbial mat, and the community awakens at a very low temperature and light conditions. Only a few groups of psychrophiles can be metabolically active, and as long as the ice remains, this community enjoys optimal conditions. When temperatures rise, cells differentiate into resistant spores (Hoham et al., 2008). This study was conducted when the ice and snow completely melted out, and not many chlorophytes were found in the microbial mat, despite being the group with the highest relative abundance of 18S rRNA sequences; therefore, they could not be physically separated and thus included directly in the study. The low abundance of green algae in the community, as well as the dominance of Nostocaceae versus other filamentous cyanobacteria (Figure 2), compared to previous studies in the same area (Fernández-Valiente et al., 2007; Rochera et al., 2013; Velázquez et al., 2017), suggests the seasonality during the ice-free period of the community of primary producers, as has been published previously (Velázquez et al., 2011). Even so, we consider that a portion of POM δ^13^C‰ signal comes from Chlorophytes. The abundance of Nostocaceae could also represent relevant differences in terms of N source, because of the potentiality of N_2_-fixation by this family (Fernández-Valiente et al., 2007). However, N_2_-fixation relevance was not evident from the data of natural abundance of the N isotopes. According to our results, tardigrades act as the main grazers of the microbial mat, feeding mainly on diatoms and green algae upon their presence in the community. Rotifers seems to feed preferentially on POM fraction between 0.5 and 5 μm, due to its size and mastax anatomy observed in the analyzed specimens. Considering that this is the estimated size for bacteria and some microeukaryotes, it seems reasonable to consider that rotifers would feed on the small POM fraction with high content in detritus and bacteria, also related to dead organic matter as has been previously described for Antarctic bdelloids specimens (Iakovenko et al., 2015). The ^13^C enrichment differences between tardigrades and rotifers (Figure 5) associate consumers of the second trophic level to multiple C sources and diet preferences, as it has been previously described in mats of maritime Antarctica (Velázquez et al., 2017), and the model plots them in different positions within the trophic network.

According to the Bayesian mixing model, nematodes would be related trophically to cyanobacteria and DOC in 42 and 21%, respectively (Figure 5). In a cyanobacteria-based microbial mat, most part of DOC is derived from cyanobacterial exudates and/or EPS (Velázquez et al., 2017). So, we conclude that nematodes might feed mostly on cyanobacteria, also considering cyanobacteria as a direct driver of the food web in the microbial mats on Byers Peninsula. Bactivorous nematodes are common in soils (Nielsen et al., 2011; Shaw et al., 2018; Caruso et al., 2019) and microbial mats from Antarctica (Velázquez et al., 2017), suggesting that by feeding on cyanobacteria, nematodes obtain proteins that would compensate for the lack of polyunsaturated fatty acids in their diet (Gaudes et al., 2006). But also, nematodes probably feed on rotifers and tardigrades, as shown by the enrichment peak along the time frame of the study. The theoretical trophic enrichment of 3.4‰ for ^15^N (Peterson and Fry, 1987) between nematodes and rotifers are almost fulfilled, and the Bayesian mixing model relates them trophically. For tardigrades this enrichment is not so evident, and nematodes only increase its δ^15^N around 2‰. Even so they appear related, and this could be due to the variability of isotopic signals in the ecosystem, with differences even between tissues of the same organism. Protozoa may also be a possible food source (Bamforth et al., 2005), but the difficulty in collecting enough biomass for isotope measurement did not allow them to be included in this study. We can expect some bias in dietary proportions, considering that some carbon sources have not been included in the model, and TEF values were made without taking into account the dietary variability within consumers. Assuming this uncertainty in some aspects of the analysis, as well as the caveats of isotopic mixing models, we are simplifying trophic relationships as much as possible to define a baseline for future studies. So, according to our isotope and modeling results, nematodes in the microbial mat from Byers Peninsula should be considered as omnivores, feeding on bacteria and other consumers. With these feeding habits and attending to their trophic position in the microbial mat food web, nematodes play a key role as top consumers of the community, connecting the two described carbon inputs into the ecosystem. In addition, by feeding on cyanobacteria and DOC, they would also contribute to the decomposition of the organic matter accumulated in these microbial mats.

Cyanobacterial mats from Byers Peninsula accumulate high standing-stocks of carbon (Velázquez et al., 2017), but its proportion had never been estimated. According to our results, during the study almost 90% of the accumulated organic carbon in the microbial mat would not be part of any of the studied trophic levels. Considering that the accumulated biomass per unit area is two to three-fold less in drylands, like Antarctica, compared to temperate ecosystems (Bay et al., 2018), this represents a huge carbon stock. This accumulated biomass, mostly EPS, represents several seasons of growth (Vincent and Howard-Williams, 1986), and is considered as an ecological adaptation of the microbial mat community to overcome fluctuating conditions across seasonal scales (Moyer et al., 1994), providing protection against temperatures and desiccation (De Los Ríos et al., 2014).

Microbial mats from Byers Peninsula have been described as a self-contained ecosystem with extremely low matter inputs (Velázquez et al., 2017). Therefore, and considering the large amount of accumulated biomass, the microbial- and myco- loops should be key to system functioning (Velázquez et al., 2016). Fungi as members of the decomposer community have an important role in the maintenance of these ecosystems. The relationship of the fungi with the POM <30 μm (Figure 5), where a large proportion of the susceptible matter to be decomposed would be concentrated, confirms their role as decomposers within the community. Sequences related to fungi (mainly Ascomycota and Basidiomycota affiliated ribotypes) represented on average 13% of the OTUs assigned to eukaryotes in this study with high-throughput sequencing. However, only 0.8% has been reported in previous studies by using different approaches (Velázquez et al., 2016). In addition, if we estimate total dry weight from calculated fungal biomass, we determined the fungal community is 10 times higher than reported in our previous study of microbial mats from Byers Peninsula, and they approximate to those areas defined as “blighted patches” (Velázquez et al., 2016), where fungal community seem to be triggering the decay of the community. Thus, an intermediate scenario is proposed, where the relative abundance of fungi would be higher than that considered in other studies, despite not being truly cold adapted (Ruisi et al., 2007). Their role as decomposers of organic matter, in a system where most of the biomass accumulates outside the main trophic groups, makes its study a key element to understand the functioning of these microbial ecosystems. Even so, a more detailed study on the relative abundance and ecological role of fungi in Antarctic microbial mats is required, taking into account the potential limitations of the analyses carried out in this study.

Antarctica is not biologically isolated, and global change has the potential to allow the establishment of diverse new species (Fraser et al., 2018). Understanding trophic positions and biotic interactions is critical for predicting future changes in species distributions and interactions. We have verified how the food web of the microbial mat from Byers Peninsula has at least four trophic levels, and each of the studied organism seems to have a specific role, with a low redundancy in ecosystem function that would be a consequence of the low diversity in these high latitudes. Nematodes have shown a key ecological role within the community, connecting the two organic carbon inputs described during the study. The presence of liquid water becomes the limiting factor for Antarctic microbial mats, and only during a few weeks in the austral summer, temperatures allow ice-free conditions. Carbon flows through the different trophic levels studied have been completed during the study, moving from primary producers to top consumers. During mid-January the environmental conditions are the most propitious for the microbial mat community, and they could be in an optimal metabolic stage (Velázquez et al., 2017). So, time intervals showed in the study could represent the fastest one, likely increasing toward the end of the summer season. Future studies are necessary to deepen the functioning and evolution of these ecosystems and their resilience to physicochemical changes.

## Author Contributions

PA, DV, ER, AJ, and AQ designed the experiments. DV, AJ, ER, and AQ collected the samples. PA analyzed the experimental data and wrote the initial manuscript. All authors contributed to elaborate the final manuscript.

## Conflict of Interest Statement

The authors declare that the research was conducted in the absence of any commercial or financial relationships that could be construed as a potential conflict of interest.

## Supplementary Material

Figure S1 Rarefaction curves of bacterial and eukaryotic community at family taxonomic level of the studied microbial mat from Byers Peninsula, Antarctica.
