# Supplementary figures and images for: Carbon Pathways Through the Food Web of a Microbial Mat From Byers Peninsula, Antarctica
Source: Front Microbiol. 2019 Mar 28;10:628. doi: 10.3389/fmicb.2019.00628 (PMC6447660; doi:10.3389/fmicb.2019.00628)

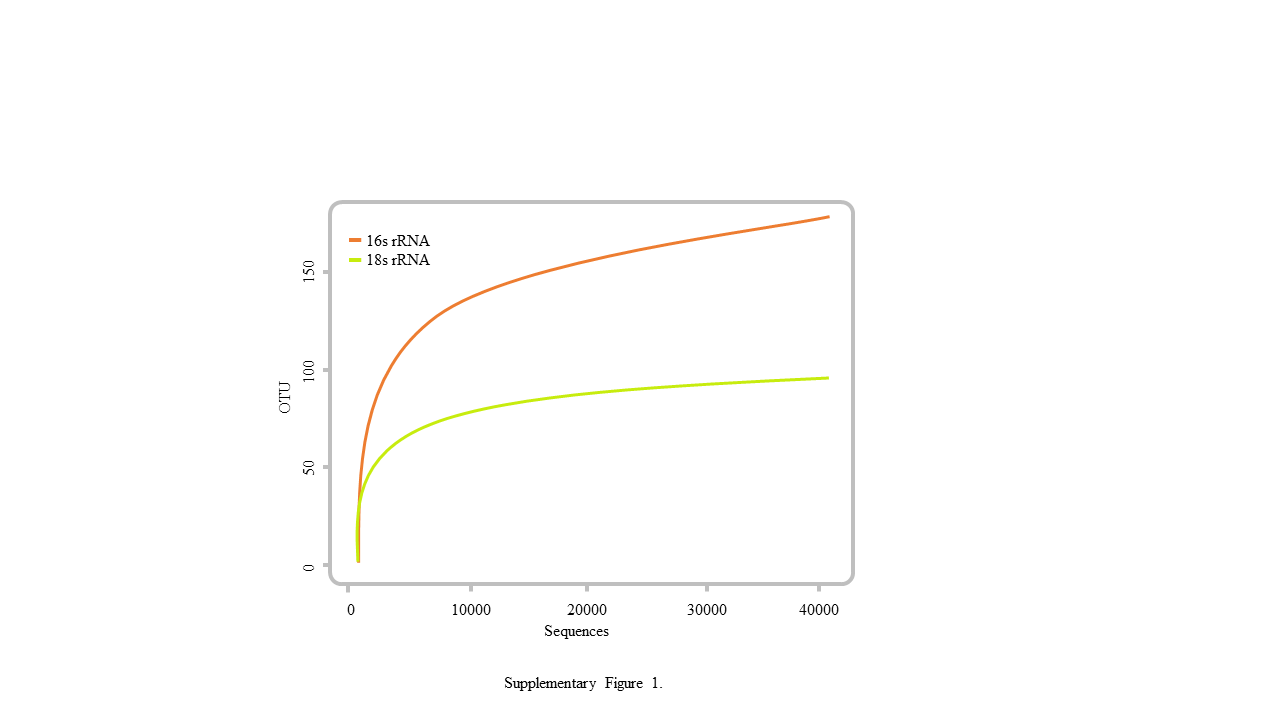

Supplement: Figure S1 — Rarefaction curves of bacterial and eukaryotic community at family taxonomic level of the studied microbial mat from Byers Peninsula, Antarctica. [file Image_1.TIF]
